# Microbial dysbiosis and associated disease mechanisms in maternal and child health

**DOI:** 10.1128/iai.00179-25

**Published:** 2025-07-01

**Authors:** Umer Ahmed, Furrmein Fatima, Hafiza Amna Farooq

**Affiliations:** 1 Institute of Microbiology, Faculty of Veterinary Science, University of Agriculture 66724, Faisalabad, Punjab, Pakistan; 2 Department of Biotechnology, Faculty of Science and Technology, University of Central Punjab 66901, Lahore, Punjab, Pakistan; 3 Department of Biochemistry, Faculty of Sciences, University of Agriculture Faisalabad 66724, Faisalabad, Punjab, Pakistan; University of California Davis, Davis, California, USA

**Keywords:** microbial dysbiosis, maternal microbiome, gut-vagina axis, pregnancy complications, neonatal infections, immune dysregulation, gut-brain axis, microbiota interventions, precision medicine, oxidative stress

## Abstract

Maternal and infant microbiome dysbiosis is associated with poor health outcomes—gut, vaginal, and placental microbiome disruptions in the gut, vaginal, and potentially placental microbiomes—though the existence of a distinct placental microbiome remains controversial—have been linked to pregnancy difficulties and neonatal infections. Dysbiosis leads to inflammation, oxidative stress, and disruptions in the gut-brain axis, which in turn affect immunological control, metabolic balance, and neurodevelopment. Maternal age, antibiotic exposure, and cesarean delivery increase microbial imbalances, raising the risk of preterm birth, gestational diabetes, and neurodevelopmental disorders. New research highlights the importance of systems-level microbial interactions in the gut-vagina axis and maternal-fetal health. Probiotics, prebiotics, and microbiota transplants may treat microbiome disorders. To reduce dysbiosis risks, research should focus on microbiome-based biomarkers, predictive AI models, and global health policy. Understanding microbial interactions at the system level is essential for maternal and child health.

## DYSBIOSIS AS A GLOBAL HEALTH CHALLENGE

A growing number of researchers identify microbial dysbiosis, which describes an imbalance in the human microbiome, as crucial to maternal and child health outcomes (1). Recent evidence highlights microbial dysbiosis as a critical factor influencing maternal and child health outcomes. The gut microbiota, along with other elements of the human microbiome, plays a fundamental role in shaping immune development, metabolic control, and brain growth (2). The adverse health effects stem from modifications made to the microbial composition that result from contemporary lifestyles, antibiotic misuse, environmental elements, and modern influences (3).

The United Nations Sustainable Development Goals prioritize efforts to reduce deaths during motherhood and infancy and address communicable and non-communicable diseases due to their importance in Sustainable Development Goals through 3.4 (4). New research shows that dysbiosis creates pregnancy complications as well as affects neonatal infections and leads to lasting health problems in children (5). This growing evidence underscores the need to integrate microbiome health into global maternal and child health policies (6).

## RELEVANCE TO MATERNAL AND PEDIATRIC DISEASES

An irregular microbial makeup leads to significant healthcare problems that influence both maternal outcomes and child health during growth and increase disease risk in the future (7). The entire microbiome of a pregnant woman, which includes her gut bacteria and vaginal and placental microorganisms, directly influences both fetal immune system development and metabolic processes (8). The complex microbial equilibrium depends on both beneficial microbes and controls pathogenic bacteria growth because any disturbance could lead to preterm birth, plus gestational diabetes and preeclampsia (9).

Immune tolerance development and protection against infections require the necessary microbial colonization processes, which begin at birth and continue throughout early infancy (10). Infants born through cesarean section receive a different microbiome from vaginally delivered infants, resulting in elevated vulnerability toward allergic conditions, asthma, and metabolic diseases (11).

The different bacterial makeup in infants born through c-section results in elevated vulnerability toward allergic conditions, asthma, and metabolic diseases (12). The diversity of gut microbes during infancy is disrupted by various infant care factors, including formula feeding, antibiotics, and environmental contaminants, which can lead to childhood obesity, inflammatory bowel disease, and neurodevelopmental disorders (13, 14). For instance, early-life gut microbiota alterations correlate with childhood obesity through changes in nutrient absorption and inflammation (13). Similarly, dysbiosis is linked to inflammatory bowel disease and neurodevelopmental disorders like autism spectrum disorder (ASD), though causal relationships remain under investigation in humans (15, 16).

## LINKAGE OF DYSBIOSIS TO DISEASES

This review aims to explore the disease mechanisms associated with microbial dysbiosis in maternal and child health. Research indicates that dysbiosis can

1. Induce immune dysregulation—dysbiosis is linked to an imbalance between pro-inflammatory and anti-inflammatory microbial metabolites, which may contribute to persistent low-grade inflammation and increased susceptibility to autoimmune diseases (17).
2. Disruption of metabolic balance—changes in the gut microbiome of both mother and infant affect nutrient metabolism, insulin sensitivity, and energy regulation, playing a role in obesity and metabolic disorders (13, 18). For example, early-life dysbiosis correlates with childhood obesity through altered nutrient absorption (19).
3. Influence neurodevelopment through the gut-brain connection—dysbiosis is associated with altered synthesis of neuroactive compounds and short-chain fatty acids, which may influence cognitive function and increase the risk of neurodevelopmental disorders, such as ASD and attention-deficit/hyperactivity disorder (15, 20). For instance, studies have identified distinct gut microbiota profiles in ASD patients, but causal mechanisms in humans remain unclear (21). Similarly, dysbiosis is linked to asthma risk, particularly in early life, though causal relationships require further investigation (22).

**Fig 1:**
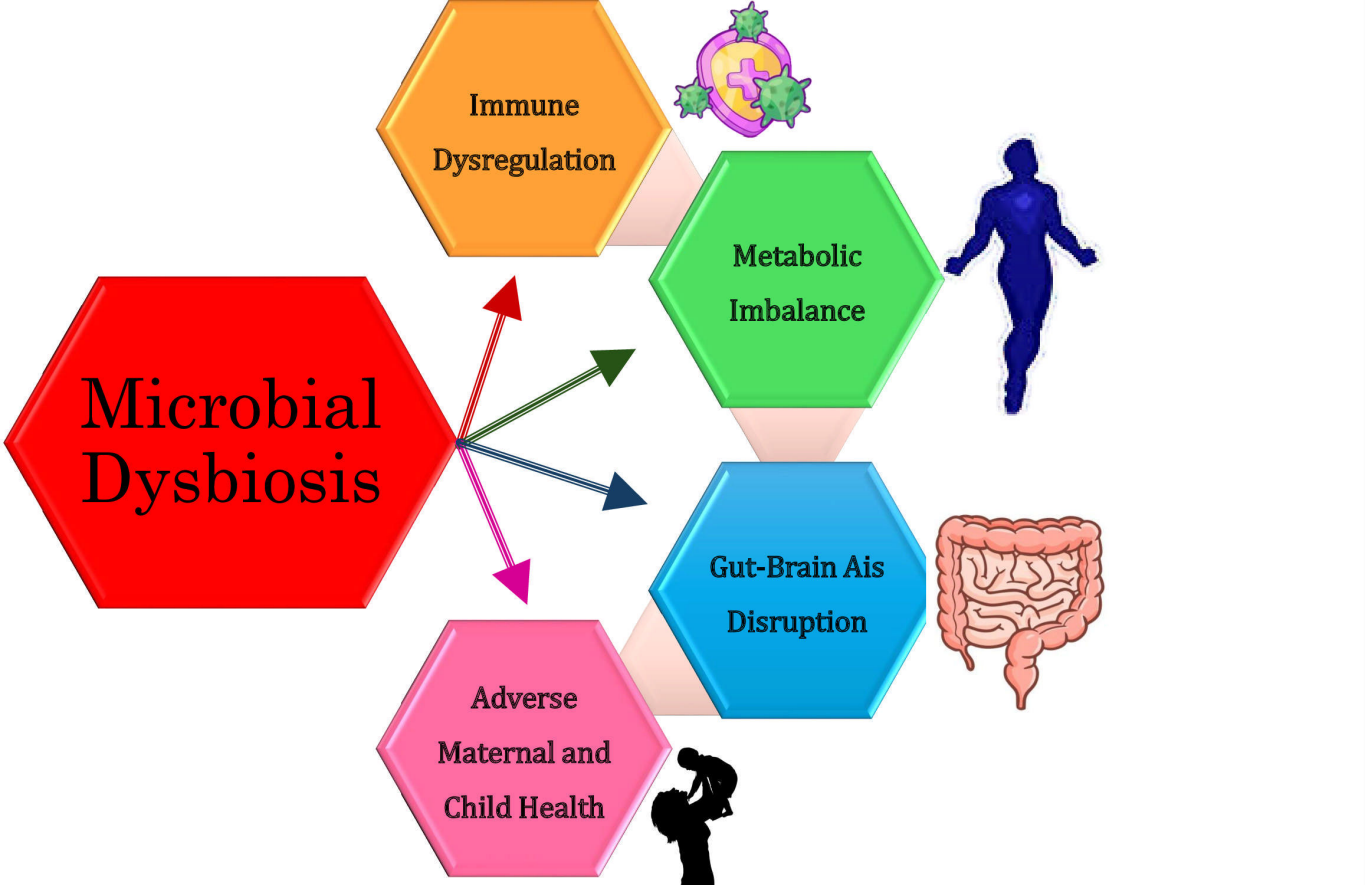
Simplified pathway illustrating the associations between microbial dysbiosis and disease mechanisms in maternal and child health.

This review aims to clarify these mechanisms and emphasize potential interventions targeting the microbiome, such as probiotics, prebiotics, fecal microbiota transplantation, and precision microbiome therapeutics, which may mitigate the adverse effects of dysbiosis on maternal and child health. The pathways linking dysbiosis to adverse health outcomes are summarized in Fig. 1.

## DYSBIOSIS IN MATERNAL AND CHILDBIRTH

Advanced maternal age (AMA) is defined as a pregnancy in a woman older than 35 years of age. AMA increases the risk for both maternal and neonatal complications, including miscarriage and stillbirth (23). Research on the specific contribution of the maternal microbiome to pregnancy outcomes and offspring behavior, conducted by recolonizing young female mice with an aged female microbiome before pregnancy, suggests that pre-pregnancy colonization of young dams with the aged microbiome significantly increases fetal loss. This indicates that the composition of the maternal gut microbiome can have a detrimental impact on pregnancy outcomes. Offspring from dams with an aged microbiome exhibited significant changes in neurotransmitters and metabolites in both the blood and the brain (24). The study indicated that age-related changes in the maternal gut microbiome can lead to chronic alterations in the behavior and physiology of offspring (25). Bacterial vaginosis (BV) during pregnancy is a significant risk factor for preterm birth and other preterm pregnancy complications. BV exposure during gestation is linked to worse neonatal outcomes, even in infants delivered at full term (26). Preeclampsia is a predominant cause of maternal and neonatal morbidity and mortality globally (27). Preeclampsia, a complication of pregnancy characterized by hypertension and organ dysfunction, is associated with gut microbiota dysbiosis, characterized by reduced bacterial diversity and enrichment of opportunistic pathogens, which may contribute to inflammation, endothelial dysfunction, and placental damage (28, 29). For example, studies have shown that patients with pre-eclampsia exhibit distinct gut microbiota profiles, but whether dysbiosis directly causes these complications requires further human mechanistic studies (29).

Microbial shifts and changes in microbial diversity play an important role in maternal health. A study involved a cohort of 2,313 pregnant women, focusing on the vaginal microbiota and its relationship with pregnancy outcomes. A study reveals that *Gardnerella vaginalis*, commonly linked to bacterial vaginosis, was detected in 77% of samples and accounted for approximately 10% of the total reads. The study found that the overall diversity of the vaginal microbiota was higher in samples from women at risk of preterm delivery (30). Table 1 summarizes the key factors contributing to microbial dysbiosis and their associated health outcomes in maternal and child health.

**TABLE 1:** Key factors contributing to microbial dysbiosis in maternal and child health

Factor	Mechanism of dysbiosis	Associated health outcomes	References
Advanced maternal age	Altered gut microbiome composition, reduced microbial diversity	Increased fetal loss, neurobehavioral changes in offspring	(31)
Antibiotic exposure	Disruption of beneficial microbes, overgrowth of pathogens	Preterm birth, neonatal infections, metabolic disorders	(32)
Cesarean delivery	Lack of vaginal microbial transfer, altered infant microbiome	Higher risk of allergies, asthma, obesity	(33)
Bacterial vaginosis	Overgrowth of pathogens (e.g., *Gardnerella vaginalis*)	Preterm birth, neonatal morbidity	(34)
Environmental contaminants	Shift in microbial balance, reduced beneficial species	Childhood obesity, inflammatory bowel disease	(35)

Another study reveals that the multi-omics approach and analysis can discover changes in gut microbiota and metabolites during pregnancy that impact immune function. The researchers performed correlation analyses and concluded that there was a marked reduction in pro-inflammatory cytokine levels in pregnant women. The study revealed that bacteria within the same microbial modules had consistent effects on cytokine levels. This suggests that these bacteria may function collectively, influencing immune responses rather than individually (36).

## DISEASE MECHANISMS AND CONSEQUENCES

### Inflammation, Oxidative Stress, and Immune Dysregulation

Microbial dysbiosis destroys the balanced interactions between microbiota and hosts, which sets off inflammation and oxidative stress processes that bring about maternal and child health difficulties (37). During pregnancy, immune regulation strongly depends on the maternal gut and vaginal microbiomes (38). The inflammation caused by dysbiosis leads to overproduction of pro-inflammatory cytokines like IL-6, TNF-α (alpha), and IL-1 that result in preterm birth and preeclampsia as adverse pregnancy effects (39).

The process of oxidative stress develops from excessive production of reactive oxygen species to worsen tissue damage, causing endothelial dysfunction, which in turn leads to gestational complications (40). The developmental problems of the fetal immune system, triggered by gut dysbiosis, impede neonatal resistance against future allergies, as well as autoimmune diseases and metabolic disorders (41).

## IMPACTS ON PREGNANCY OUTCOMES AND CHILD DEVELOPMENT

Abnormalities in maternal microbial populations affect both maternal health outcomes during pregnancy and the development of child health. Research indicates that maternal dysbiosis causes preterm labor and leads to an infant’s low birth weight, together with an increased risk for neonatal infections (42, 43). Scientific research indicates that decreased Lactobacillus species combined with excessive pathogen growth in the vaginal microbiome elevates risks for bacterial vaginosis, pregnancy failure, and pregnancy complications (44).

Long-term microbial dysbiosis causes adverse effects that damage both neurodevelopmental well-being and metabolic processes, as well as immune system health (45, 46). Delayed neural communication resulting from gut-brain axis problems makes newborn children at risk for developing asthma alongside obesity and ASD (47). Early microbial colonization, which occurs because of cesarean delivery and antibiotic exposure, leads to higher probabilities of developing allergic diseases and metabolic syndromes in infants (48).

## NOVELTY: SYSTEM-LEVEL INTERACTIONS (E.G., GUT-VAGINA AXIS)

The study of dysbiosis brought forward the system-wide microbial relationships, including the gut-vagina axis, which explains maternal microbiome connectivity as a novel approach (49). The composition of vaginal microorganisms experiences potential modifications from maternal pregnancy outcomes because gut dysbiosis appears to regulate immune signaling and microbial metabolite exchanges (50). If this axis becomes disrupted, then pregnant women are at higher risk for bacterial vaginosis and intrauterine infections. Research demonstrates that improving maternal health requires a comprehensive strategy for intervention initiatives (51).

The development of treatment approaches can be based on microbiome science, following the establishment of a better understanding of these complex biological relationships. Maternal health professionals use specific probiotics combined with microbiota transplants alongside dietary interventions to restore microbial balance and decrease sickness threats for both mothers and their children (52).

## FUTURE DIRECTIONS

In conclusion, precision medicine strategies demonstrate significant potential to minimize dysbiosis-related health damage in pregnant women and infants (53). The discovery of microbiome-specific treatments, such as individualized probiotics, as well as microbiome transplants and customized dietary approaches, offers new pathways to restore microbial balance (54). Artificial intelligence (AI) integrated with systems biology is increasingly applied to predict disorders associated with dysbiosis (55). Machine learning models can analyze microbial community dynamics, predict outcomes like preterm birth, and even guide personalized microbiome therapeutics by identifying microbial biomarkers (55, 56). Research collaborations across global institutions need to work together to address microbial science inequalities between regions and develop multi-area, region-specific interventions (57). Future research priorities should aim to identify microbiome markers for disease forecasting, while also making microbiome management a critical component of healthcare strategies related to maternal and pediatric care (58). Table 2 outlines potential microbiome-based interventions to mitigate dysbiosis-related health risks in maternal and child populations.

**TABLE 2:** Potential microbiome-based interventions, their mechanisms of action, and benefits for maternal and child health

Intervention	Mechanism of action	Potential benefits	References
Probiotics	Reintroduce beneficial bacteria (e.g., Lactobacillus)	Reduce inflammation, improve pregnancy outcomes	(59)
Prebiotics	Promote growth of beneficial gut microbes	Enhance metabolic balance, support immunity	(60, 61)
Fecal microbiota transplant	Restore microbial diversity	Mitigate dysbiosis-related complications	(62)
Dietary modifications	Adjust nutrient intake to favor healthy microbiota	Decrease risk of obesity, neurodevelopmental issues	(63)
Precision microbiome therapies	Target specific microbial imbalances	Personalized treatment for dysbiosis	(64)

## CONCLUSION

Microbial dysbiosis plays a pivotal role in maternal and child health, with far-reaching consequences on immune development, metabolic regulation, and overall well-being. This review underscores how alterations in microbiome composition contribute to inflammatory responses, oxidative stress, and immune dysregulation, ultimately impacting pregnancy outcomes and childhood health trajectories. The gut-vagina axis, a key interaction within the microbiome, underscores the systemic nature of microbial influences on maternal-fetal health. While significant strides have been made in understanding dysbiosis, a substantial gap remains in mechanistic studies across diverse populations, particularly in low-resource settings. There is an urgent need for standardized methodologies, more extensive cohort studies, and multi-omics approaches to elucidate these complex interactions better.
